# Microbial amelioration of salinity stress in endangered accessions of Iranian licorice (*Glycyrrhiza glabra* L.)

**DOI:** 10.1186/s12870-022-03703-9

**Published:** 2022-07-05

**Authors:** Seyyed Sasan Mousavi, Akbar Karami, Mohammad Jamal Saharkhiz, Mohammad Etemadi, Mohammadhossein Ravanbakhsh

**Affiliations:** 1 Department of Horticultural Science, School of Agriculture, Shiraz University, Shiraz, Iran; 2 Institute of Environmental Biology, Ecology and Biodiversity Group, Utrecht University, Utrecht, Netherlands

**Keywords:** Antioxidant Enzymes, Azotobacter, Licorice Population, Plant Growth-Promoting Rhizobacteria, Salt Stress

## Abstract

**Background:**

*Glycyrrhiza glabra* L. is a medicinal and industrial plant that has gone extinct due to different abiotic stress caused by climate change. To understand how the plant-associated microorganism can support this plant under salinity, we collected sixteen Iranian accessions of *G. glabra* L., inoculated their rhizomes with *Azotobacter* sp. (two levels, bacterial treatments, and no-bacterial treatments, and grown them under salinity stress (NaCl levels; 0, and 200 mM).

**Results:**

Two accessions of Bardsir and Bajgah significantly showed higher resistant to salinity, for example by increasing crown diameter (11.05 and 11 cm, respectively) compared to an average diameter of 9.5 in other accessions. Azotobacter inoculation caused a significant increase in plant height and crown diameter. Among studied accessions, Kashmar (46.21%) and Ilam (44.95%) had the highest rate of membrane stability index (MSI). Evaluation of enzyme activity represented that bacterial application under salinity, increased polyphenol oxidase (PPO) (0.21 U mg^−1^ protein), peroxidase (POD) (3.09 U mg^−1^ protein U mg^−1^ protein), and phenylalanine ammonia-lyase (PAL) (17.85 U mg^−1^ protein) activity. Darab accession showed the highest increase (6.45%) in antioxidant potential compared with all studied accessions under Azotobacter inoculation. According to principal component analysis (PCA), it was found that the accession of Meshkinshahr showed a more remarkable ability to activate its enzymatic defense system under salt stress. Also, three accessions of Meshkinshahr, Eghlid, and Ilam were categorized in separated clusters than other accessions regarding various studied treatments.

**Conclusion:**

Analysis indicated that five accessions of Meshkinshahr, Rabt, Piranshahr, Bardsir, and Kermanshah from the perspective of induced systematic resistance are the accessions that showed a greater morphophysiological and biochemical outcome under salinity. This study suggested that, inoculation of with Azotobacter on selected accession can relieve salt stress and support industrial mass production under abiotic condition.

**Supplementary Information:**

The online version contains supplementary material available at 10.1186/s12870-022-03703-9.

## Background

*Glycyrrhiza glabra* L. is one of the most well-known medicinal herbs in the Fabaceae family, and its members are now extensively used as feed and food. Food additives, such as flavors and sweetening agents, are the most common industrial uses of *G. glabra* [1]. The root is used to flavor American-style tobacco, chewing gum, sweets, baked goods, ice cream, and soft drinks [2]. Glycyrrhizin, a triterpenoid saponin approximately 50 times sweeter than sucrose, is the principal active component found in roots [3, 4]. Like other plants in nature, this important medicinal plant is subjected to environmental challenges, particularly salt stress, as a result of global climate change. Because licorice has been overharvested in the wild for many years, this species is threatened with extinction, so its cultivation and domestication are urgently needed. In the present study, two methods, including Azotobacter and selection of tolerant genotypes, were evaluated to prevent the extinction of the species and improve its domestication and cultivation.

One of the most harmful environmental stresses is soil salinity. The accumulation of salts in the soil, notably sodium (Na^+^) and chloride (Cl^−^) ions, causes the salinization of agricultural lands [5]. Water conductivity, soil porosity, and aeration are all hampered by high Na^+^ accumulation [6]. Furthermore, soil salinity stress has a detrimental impact on microbial diversity within and around plant roots. When a plant is exposed to salt, it suffers many biochemical, physiological, and molecular changes that suppress its growth and development [7]. Plants' biochemical and physiological responses to salt stress change, affecting nearly all plant functions. Salinity has a substantial influence on plant development, perhaps through decreasing enzyme activity and biochemical components [8]. Cell membranes, proteins, lipids, and nucleic acids (DNA, RNA) are all damaged by reactive oxygen species (ROS) under salinity stress, as well as causing programmed cell death [9]. Because of the excessive buildup of Na^+^ and Cl^−^ ions, salinity also causes hypertonic stress [10]. To reduce the harmful effects of excessive salinity on plant development, several methods have been developed, including plant selection of resistant accessions and, more recently, the introduction of plant growth-promoting bacteria (PGPB) [11].

Agricultural practices that have been used in the past and enhanced salt-tolerant crop varieties will not be enough to achieve the yields shortly. Plants' rhizospheres are home to various microorganisms, some of which can deal with salt stress. These salinity-tolerant plant growth-promoting rhizobacteria (PGPR) help plants survive in salty environments. Furthermore, the microbiome of plants that are naturally linked with them, can defend the host through stress avoidance, tolerance, and resistance mechanisms. PGPRs are free-living bacteria found in the rhizosphere that help agricultural plants develop and yield more efficiently. Microorganisms that can thrive in, on, or around plant tissues and stimulate plant growth through a variety of methods are known as PGPB [12]. These processes and their consequences might be categorized as either direct or indirect. Biological nitrogen fixation, phosphate solubilization, mineralization, and siderophore formation [13]. Little is known of the mechanisms by which soil microbes regulate root expression of specific aquaporin isoforms, but it is tempting to speculate that microbial alteration of root hormone status and synthesis of plant hormones such as indole, cytokinins, or gibberellins are examples of natural processes linked to increased nutrient availability [14, 15]. Also, PGPRs increase the efficiency of inoculated plants to take up selections for maintaining a high K/Na ratio, decreasing plant Na^+^ accumulation by excreting EPS to bind cations (especially Na +), and change root architecture, morphology, and hydraulic conductance [16]. Despite PGPB's ability to tolerate high salinity, plant biochemical reactions must be taken into account.

To date, several efforts have been made to find benchmarks that can effectively select salt-tolerant or salt-resistant genotypes. However, the probability that the stress-tolerance genes in a plant are centralized and recognized by physiological methods is very limited. Therefore, morphophysiological and biochemical sustainability and stability under stress conditions are among the main indicators of selection for finding tolerant genotypes in many breeding programs [17]. Identification of salt-tolerant accessions can improve the growth and productivity of the plant in the salt-affected areas [18]. The formation of reactive oxygen forms (ROS) is one of the physiological reactions of plants to salt stress, which disrupts the equilibrium between ROS production and the removal of their effects by antioxidant enzymes, causing oxidative destruction [19]. Plants have several defensive apparatuses to decrease ROS to combat the harmful effects of salt stress. The change in enzymatic antioxidant activity, which works by coordinating numerous enzymes such as CAT (Catalase), POD, and SOD (Superoxide dismutase), reacting with ROS to keep it at a low level, is one of the defensive mechanisms. For the effective elimination of ROS, salt-tolerant plants have an excellent antioxidant system [20, 21]. Chlorophyll degradation and membrane lipid peroxidation are caused by ROS, which reduces membrane fluidity and selectivity [22]. Loss of chlorophyll and lipid peroxidation, as measured by malondialdehyde, and lipid peroxidation products, are indicators of oxidative damage [23]. To counteract ROS and protect cells from oxidative damage, plants have evolved various enzymatic and non-enzymatic strategies in response to salinity [24]. The detoxification of superoxide and H_2_O_2_ by antioxidant enzymes has already been identified as a key component of the plant response mechanism to salinity [25, 26]. High salinity has been shown to cause the production and accumulation of ROS in plant cells [27]. CAT is a crucial enzyme detoxifying H_2_O_2_ and is involved in scavenging H_2_O_2_ during salt stress and other abiotic stress situations [28]. Although Ascorbate peroxidase (APX) has the same general function as CAT, it uses ascorbate as a reductant to catalyze the elimination of H_2_O_2_. In higher plants, APX is a family of isozymes that regulates the intracellular amount of H_2_O_2_ [29]. Due to the increase in agricultural land affected by salt stress, overharvesting of this endangered crop, and the high and critical demand of the industry for licorice raw material, this study was conducted to evaluate salt-tolerant licorice accessions using plant growth-promoting bacteria, focusing on morphophysiological and enzymatic aspects. Azoobacter makes the plant more resistant to salinity, improves growth parameters, and increases plant productivity under salt stress by maintaining balanced nutrient cycling [30]. Also, the evaluation of germplasm responses to salinity is an essential screening technique that is required [31]. There are few studies regarding salinity stress and its effects on licorice plants. Therefore, this study investigates the morphophysiological responses of licorice accessions collected from different climatic regions of Iran in response to salinity stress under symbiosis with growth-promoting bacteria.

## Materials and methods

### Experimental design

This experiment was carried out in the greenhouse at the Faculty of Agriculture, University of Shiraz, Iran. The studied factors were consisting of two levels of *Azotobacter* sp. (no-bacterial treatment (A_0_) and bacterial treatment (A_1_)), two levels of salinity (no salinity (S_0_), and 200 mM NaCl (S_1_)), and sixteen accessions of licorice plants. ​

### Soil preparation

A mixture of arable soil and sand in a 1:1 ratio was used in disinfected plastic pots. Each pot contained 25 kg of the prepared soil. Table 1 shows the physicochemical properties of the soil used.

**Table 1:** Physiochemical properties of the soil used in the present experiment

Soil texture	Sand	Silt	Clay	OM^a^	N	Cu-DTPA	Mn-DTPA	Zn-DTPA	P-Olsen	K	Fe	EC	pH
Sandy-Clay	%					mg/kg						ds/m	-
	50	11	39	0.9	0.14	0.93	4.3	0.23	8	53	4.62	1.4	7.6

In table ^a^OM stands for organic matter

### Plant materials

**Fig. 1:**
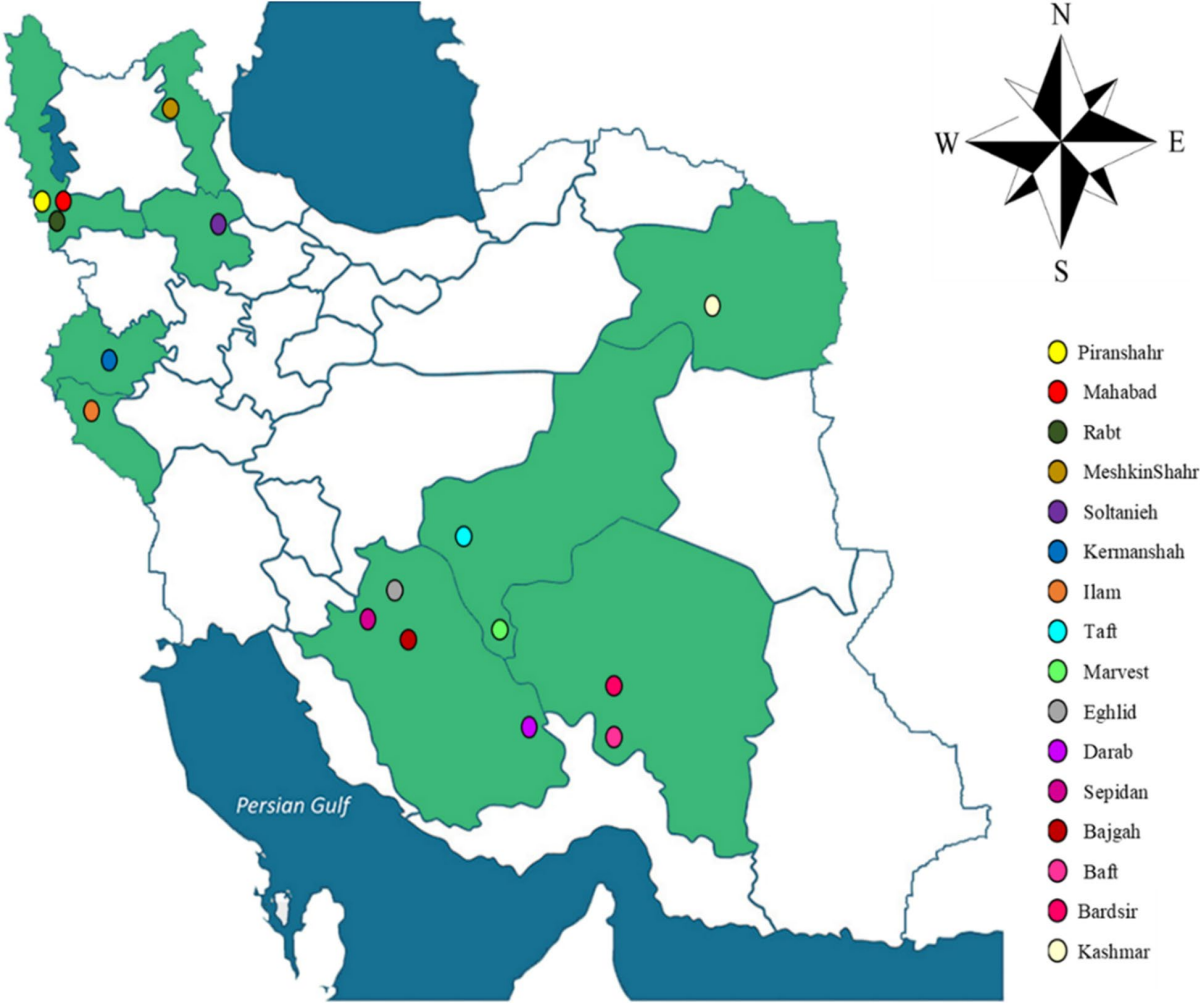
The collection areas for the investigated accessions of *G. glabra*

The rhizomes of sixteen licorice accessions were collected from wild natural resources in 16 public regions of Iran (Table 2, Fig. 1). The collection was done following national and scientific guidelines as described by Esmaeili et al. [3] and based on the International Standard for Sustainable Wild Collection of Medicinal and Aromatic Plants (ISSC-MAP) (Version 1.0) prepared by the Medicinal Plant Specialist Group of the IUCN Species Survival Commission (The World Conservation Union). The permission to collect wild plants from public natural resources is obtained from the Natural Resources and Watershed Management Organization (Iranian Government Organization). The plants' voucher specimens were deposited in the Medicinal Plants and Drugs Research Institute's herbarium at Shahid Beheshti University in Evin, Tehran, Iran (Table 2). To begin, the gathered accessions are conserved in the *ex-situ* conservation field at Shiraz University's School of Agriculture in Shiraz, Iran. A year after, adapted accessions were propagated by rhizome cuttings. The rhizomes were cut into equal fragments (15 cm) with sharp gardening scissors, dipped into fungicide solution (benomyl 1%), and then cultivated in the pots with prepared soil. A year after, the cuttings of all accessions were rooted; and then they were chosen to enter the experiment. As a result, sixty-four treatments with three duplicates were carried out, totaling 192 pots.

**Table 2:** Geographical characteristics of different *G. glabra* accessions collected from Iran

No	Accessions	Province	Longitude (E)	Latitude (N)	Altitude (m)	Voucher Number
1	Eghlid	Fars	52°29′37.9″	30˚44′ 30.8″	2319	MPH-2670–1
2	Bajgah	Fars	52°35′ 17.98″	29˚43′ 26.14″	1798	MPH-2670–2
3	Darab	Fars	54°25′ 37.64″	28˚43′ 3.95″	1081	MPH-2670–3
4	Sepidan	Fars	52°00′ 41.5″	30˚13′ 21.5″	2157	MPH-2670–4
5	Ilam	Ilam	46°17′ 43.72″	33˚40′ 49.64″	1032	MPH-2670–5
6	Baft	Kerman	56°27′ 57.6″	29˚15′ 7.1″	2241	MPH-2670–6
7	Bardsir	Kerman	56°15′ 21.94″	29˚52′ 40.41″	2338	MPH-2670–7
8	Kashmar	Razavi Khorasan	58°27′ 51.07″	35˚23′ 59.70″	1632	MPH-2670–8
9	Kermanshah	Kermanshah	46°59′ 21.37″	34˚23′ 05.91″	1371	MPH-2670–9
10	MeshkinShahr	Ardabil	47°42′ 54.80″	38˚25′ 01.10″	1412	MPH-2670–10
11	Taft	Yazd	53°50′ 59.3″	31˚39′ 44.1″	2286	MPH-2670–11
12	Marvest	Yazd	54°13′ 51.9″	30˚26′ 59.8″	1542	MPH-2670–12
13	Soltanieh	Zanjan	36˚24′ 40.21″	48°44′ 19.40″	1842	MPH-2670–13
14	Rabt	West Azarbaijan	36˚12′ 41.35″	45°31′ 54.68″	1075	MPH-2670–14
15	Piranshahr	West Azarbaijan	36˚37′ 42.95″	45°07′ 54.92″	1492	MPH-2670–15
16	Mahabad	West Azarbaijan	36˚48′ 12.68″	45°43′ 09.53″	1410	MPH-2670–16

### Bacterial solution preparation and treatments application

Bacteria from the genus *Azotobacter* sp. (endemic to Iran, from the Iranian Research Organization for Science and Technology, Persian Type Culture Collection (PTCC No: 1658)) were cultured in a nutrition broth medium on a temperature-controlled shaker for 24 h at 28 ± 2 °C. At 600 nm, the bacterial density was measured. An inoculum of 106 CFU/ml was generated by centrifuging the freshly developed bacterial culture at 10,000 × g for 5 min for inoculation with *Azotobacter* sp. The suspensions that resulted were utilized to treat licorice plants [32]. For Azotobacter treatments (A_1_), the bacterial solution was sprayed on the roots of cuttings until the roots were completely wet and the solution was dripped from them. The roots in the no-bacterial treatment (A_0_) were sprayed with distilled water. They were then cultivated in pots (25 kg) and maintained for 11 months, before salinity treatment. The salt-treated plants were irrigated every five days with a salt solution of 200 mM (S1) (To prevent osmotic shock, salt solution was added stepwise to the pots). Control and salt-treated plants were kept under saline stress in a greenhouse (16 h light, 25–28 °C, 120 µmol m-2 s-1 photon flux density, and 8 h of dark, ~ 15–21 °C for two months).

### Studied parameters

After two months, at the end of the experiment, the aerial parts and roots of the plants were harvested separately. To measure some parameters fresh leaves and roots from all pots were separated, frozen in liquid nitrogen (-96 °C), and then stored in a freezer at -80 °C. Some leaves and roots also were dried for other parameters measurements. In this experiment, various morphological and biochemical parameters including plant height, crown diameter, the activity of SOD, POD, PPO, PAL, APX, CAT enzymes, H_2_O_2_ content, antioxidant activity, electrolyte leakage (EL), leaf membrane stability index (MSI), and Malondialdehyde (MDA) were measured. Plant height and crown diameter were measured by a scientific ruler and Caliper (0.01 mm accuracy) respectively.

### Enzyme extraction and activity assay

Half a gram (0.5 g) of the fresh leaf was excised and crushed in liquid nitrogen for enzyme extraction (CAT, APX, SOD, POD, PPO, and PAL), then homogenized with 2 mL of extraction buffer (50 mmol L^−1^ potassium phosphate buffer, pH = 7, containing 2 mmol L^−1^ EDTA), and in a 2 ml Eppendorf tube with 1% polyvinylpyrrolidone (PVP) and centrifuged at 13,000 × g for 10 min at 4 °C. The supernatant was used to measure protein content and enzyme activity [33].

One unit (U) of SOD activity was defined as the amount of enzyme affecting 50% of the maximum inhibition of nitroblue-tetrazolium (NBT) reduction. The enzyme activity was presented as Ug^−1^ FW. Variations in absorbance at 470 nm were used to monitor POD activity, and activity was reported as Ug^−1^ FW [34]. Variations in absorbance at 240 nm (Ug^−1^ FW) were used to measure the activity of CAT [35]. The activity of APX was determined by measuring the reduction in absorbance at 290 nm and was represented as Ug^−1^ FW [36]. The activity of PPO was measured using Kumar and Khan's method [37]. The activity of the enzyme was measured in units of U g^−1^ FW min^−1^. The activity of PAL was estimated by measuring the absorbance of trans-cinnamic acid at 290 nm [38, 39].

### Antioxidant activity

The DPPH (2, 2-diphenyl-1-picrylhydrazyl, 95 percent, Sigma-Aldrich, Steinheim, Germany) free radical scavenging technique was used to evaluate total antioxidant activity in root extract [40]. Dry root tissue (0.5 g) was crushed and stored in 2 mL 70% ethanol on the shaker for 24 h. The antioxidant activity of the filtered extract was determined. The obtained extract was then combined with 100 mL DPPH (0.1 mM in methanol). The mixture was mixed and stored at ambient temperature for 30 min in the dark. A microplate Spectrophotometer (Model Epoch Biotech, Germany) was used to measure the mixture absorbance at 517 nm.

### Electrolyte leakage, membrane stability index, MDA, and H_2_O_2_ assay

The percentage of electrolyte leakage was used to measure membrane permeability using the method of previous research [33, 41, 42]. The leaves were chopped and put in separate stopper vials holding 10 ml of distilled water after being cleaned three times with distilled water to eliminate surface contamination. On the shaker, these samples were incubated at room temperature for 24 h. A conductivity meter (Model Ohm-419) was used to test the solution's electrical conductivity (EC1) after incubation. After cooling the bathing solutions to room temperature, the samples were put in an autoclave at 100 °C for 20 min. The second measurement EC2) was taken after cooling the bathing solutions to room temperature. The EL and MSI were computed as follows:

$$\mathrm{EL}= (\mathrm{EC}1/\mathrm{EC}2) \times 100\mathrm{ and MSI}= 1-(\mathrm{EC}1/\mathrm{EC}2) \times 100$$

To measure the malondialdehyde (MDA) quantity, 0.5 g of the fresh leaf was crushed in trichloroacetic acid (TCA) (1%, 2 mL) and centrifuged at 10,000 × rpm (10 min). The supernatant (250 µL) was mixed with 1 mL of 20% TCA containing 0.5% thiobarbituric acid (TBA). The solution was kept in hot water at 90 °C for 30 min, before being cooled promptly in an ice bath and centrifuged. A microplate reader spectrophotometer read the absorbance at 532, 600, and 450 nm (Epoch Biotek, Winooski, VT, USA). The supernatant absorbance was determined at 450, 532, and 600 nm, respectively [43].

Fresh leaves (0.5 g) were homogenized in trichloroacetic acid (TCA) (1%) (w/v) (2 mL) and were centrifuged at 10,000 × g for 10 min at 4 °C. The supernatant (250 µL) was mixed with 100 mmol L^−1^ phosphate buffer (250 µL) (pH = 7), together with 1 mol L^−1^ potassium iodide (KI) (500 µL) in a 2 ml Eppendorf tube. After thoroughly vortexing the solution, its absorbance was read at 390 nm using a microplate reader spectrophotometer (Epoch Biotek, Winooski, VT, USA). The content of H_2_O_2_ was determined with a standard curve of H_2_O_2_ [44].

### Statistical analysis

The Minitab software (version 18) was used to conduct the statistical analysis. The experiments were organized in a completely randomized design (CRD) based on a factorial experiment with three replications. The factors were licorice accessions, salt levels, and bacterial inoculation. Analysis of variance (ANOVA) followed by Tukey's multiple range test (p ≤ 0.05) was used to compare treatment means. In the case of significant interactions, the slice method was used for mean comparisons. The slice method selects a subset of elements (part of a larger set) based on the overall index. This method is used to limit the selection of elements in a group, by a start and endpoint. Minitab software (version 17) was used to perform principal component analysis (PCA) based on a correlation matrix and draw a dendrogram based on Euclidean distance. Corrplot was created using R software version 4.1.1 (Corrplot package).

## Results

### Crown diameter and plant height

**Fig. 2:**
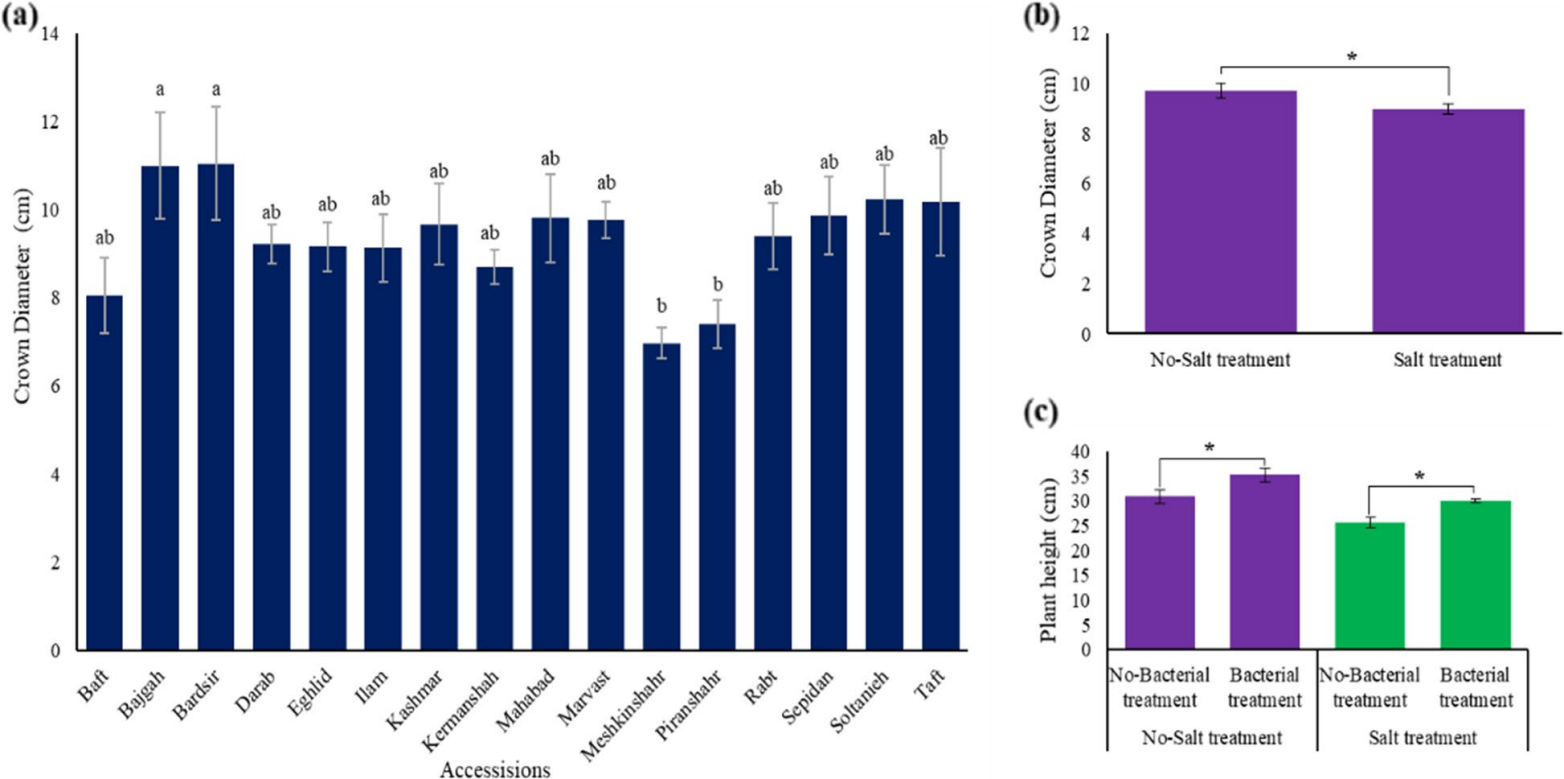
Crown diameter variation of Iranian licorice under studied treatments. **a** crown diameter variation in different studied accessions **b** crown diameter variation under salinity stress. According to the analysis of variance that only the main effect of **a** accession and **b** salinity levels showed a significant difference on crown diameter, just their mean comparisons are shown. **c** According to the analysis of variance that double effects of Azotobacter and salinity levels showed a significant difference on plant height, the slice method was used for mean comparisons. Columns with the same letter are not significantly different (*p* ≤ 0.05) (Tukey test). Bars stand for standard error (SE)

The results of the present study showed that different accessions of Iranian licorice exhibited significant (*p* ≤ 0.05) variations in their crown diameters. Regarding this growth parameter, two accessions of Bardsir and Bajgah had higher values, 11.05 and 11 cm, respectively (Fig. 2a), which could indicate their better tolerance to salt stress. Compared to all accessions, Meshkinshahr and Piranshahr, as a sensitive accessions to salt stress, showed a minimum crown diameter (Fig. 2a) that may result from more growth inhibition under salinity stress. Also, in the present study, the salinity stress significantly could reduce crown diameter by about 7.5% toward control treatments (no salinity) (Fig. 2b). As well, results showed that Azotobacter inoculation caused a significant (*p* ≤ 0.05) increase in plant height (35.23 cm) (Fig. 2c). In this term, Azotobacter inoculation integrated with salinity prevents plant height reduction of the studied licorice accessions.

### Electrolyte leakage and leaf Membrane stability index

**Fig. 3:**
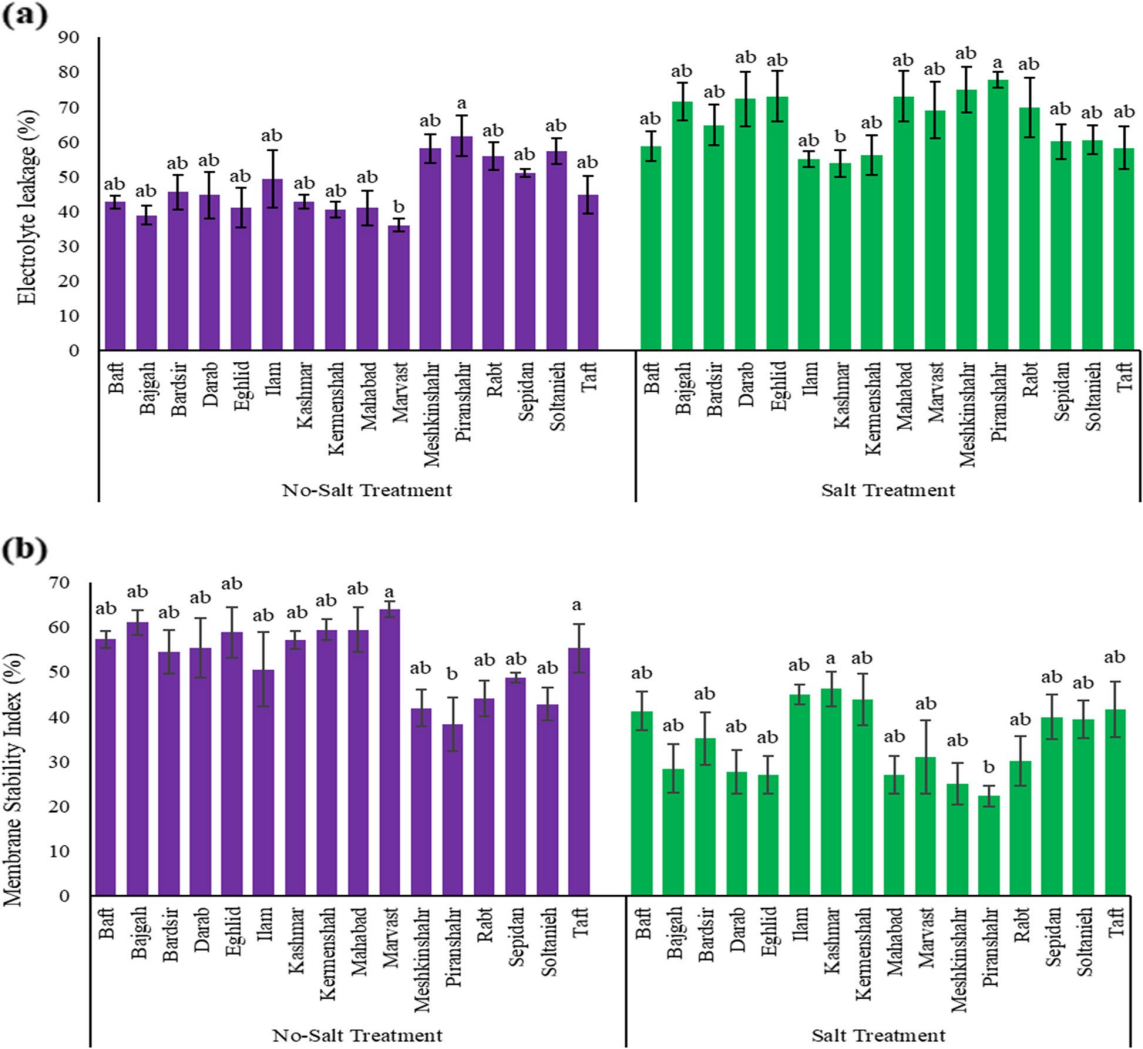
**a** Electrolyte leakage and **b** membrane stability index variation of Iranian licorice under salinity stress. According to the analysis of variance that double effects of accessions and salinity levels showed a significant difference, the slice method was used for mean comparisons. Columns with the same letter are not significantly different (*p* ≤ 0.05) (Tukey test). Bars stand for standard error (SE)

The percentage of electrolyte leakage was measured as an indicator in assessing the health of plant cell membranes. In the present study, the amount of electrolyte leakage of plant cells increases with increasing salinity among different accessions. Comparing electrolyte leakage among all investigated accessions showed that the most tolerant accession is Kashmar (53.79%), and the most sensitive to salt stress was related to the accession of Piranshahr (77.67%) (Fig. 3a). Then, according to the results of electrolyte leakage, the membrane stability index was measured. The results showed that the two accessions of Kashmar (46.21%) and Ilam (44.95%) had the highest rate of this index (Fig. 3b).

### H_2_O_2_, MDA, and antioxidant activity assay

Examining the amount of H_2_O_2_ produced in different accessions of Iranian licorice under the interaction of Azotobacter inoculation and salinity, the highest content of H_2_O_2_ production in different treatments belongs to the Bardsir accession (17 nmol/g _FW_) (Table S1). By application of Azotobacter, the highest reduction in H_2_O_2_ quantity was detected in Mahabad accession (76.9% decrease) under salinity stress toward A_1_S_0_. The findings of the present study (Table S1) indicated that the maximum content of MDA belonged to the accession of Meshkinshahr (685.41% increase compared with the control). It indicates the highest sensitivity of this accession to salinity stress; however, it was adjusted by the use of Azotobacter application and the amount of MDA just showed a 34.79% increase toward its control (2.4 µmol/g _FW_). Antioxidant activities of different accessions of Iranian licorice were increased, by salinity stress. The highest increase in antioxidant activity was recorded in Kashmar accession (8.47%). But when salinity stress was associated with Azotobacter inoculation, licorice accessions showed different responses in terms of antioxidant activity. For instance, under bacterial inoculation, the Darab accession can be mentioned as the accession with the highest percentage of increase (6.45%) in antioxidant activity and the Mahabad accession with the highest percentage of decrease (9.79%) in antioxidant activity compared with other accessions.

### Enzyme assay

**Fig. 4:**
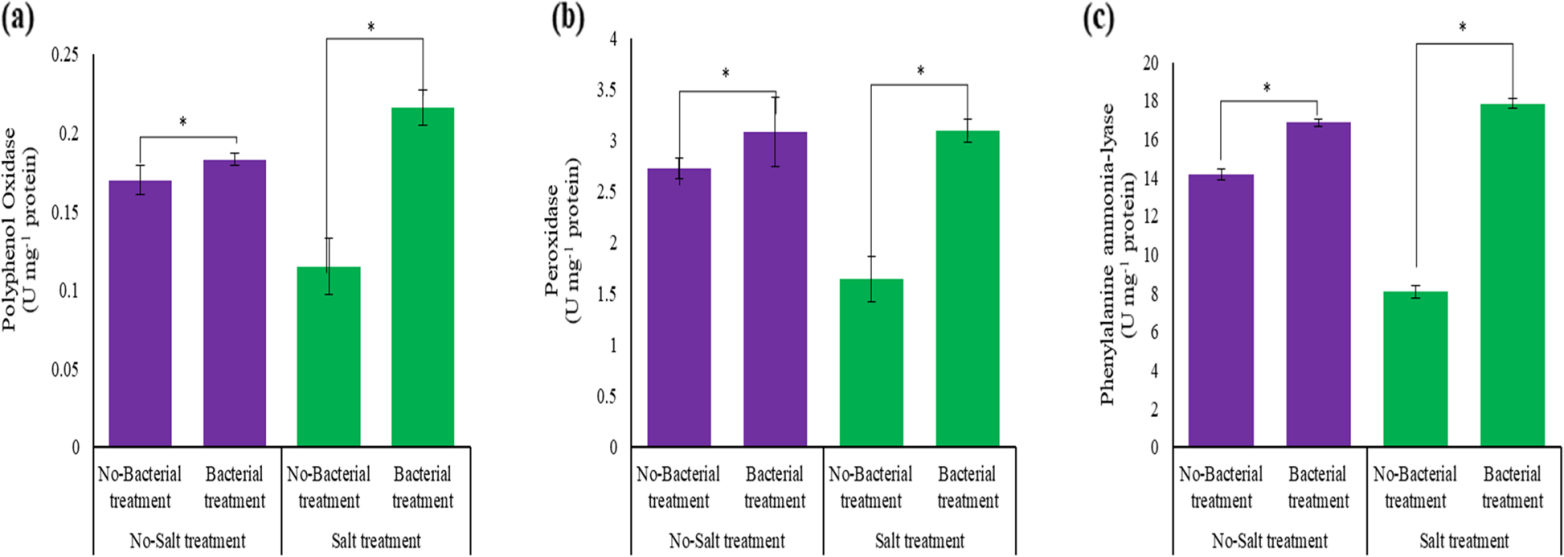
The enzyme activity of Licorice under integrated salinity stress and Azotobacter application. **a** Polyphenol oxidase, **b** Peroxidase, and **c** Phenylalanine Ammonia-Lyase. According to the analysis of variance that double effects of Azotobacter and salinity levels showed a significant difference, the slice method was used for mean comparisons. Columns with the same letter are not significantly different (*p* ≤ 0.05) (Tukey test). Bars stand for standard error (SE)

**Fig. 5:**
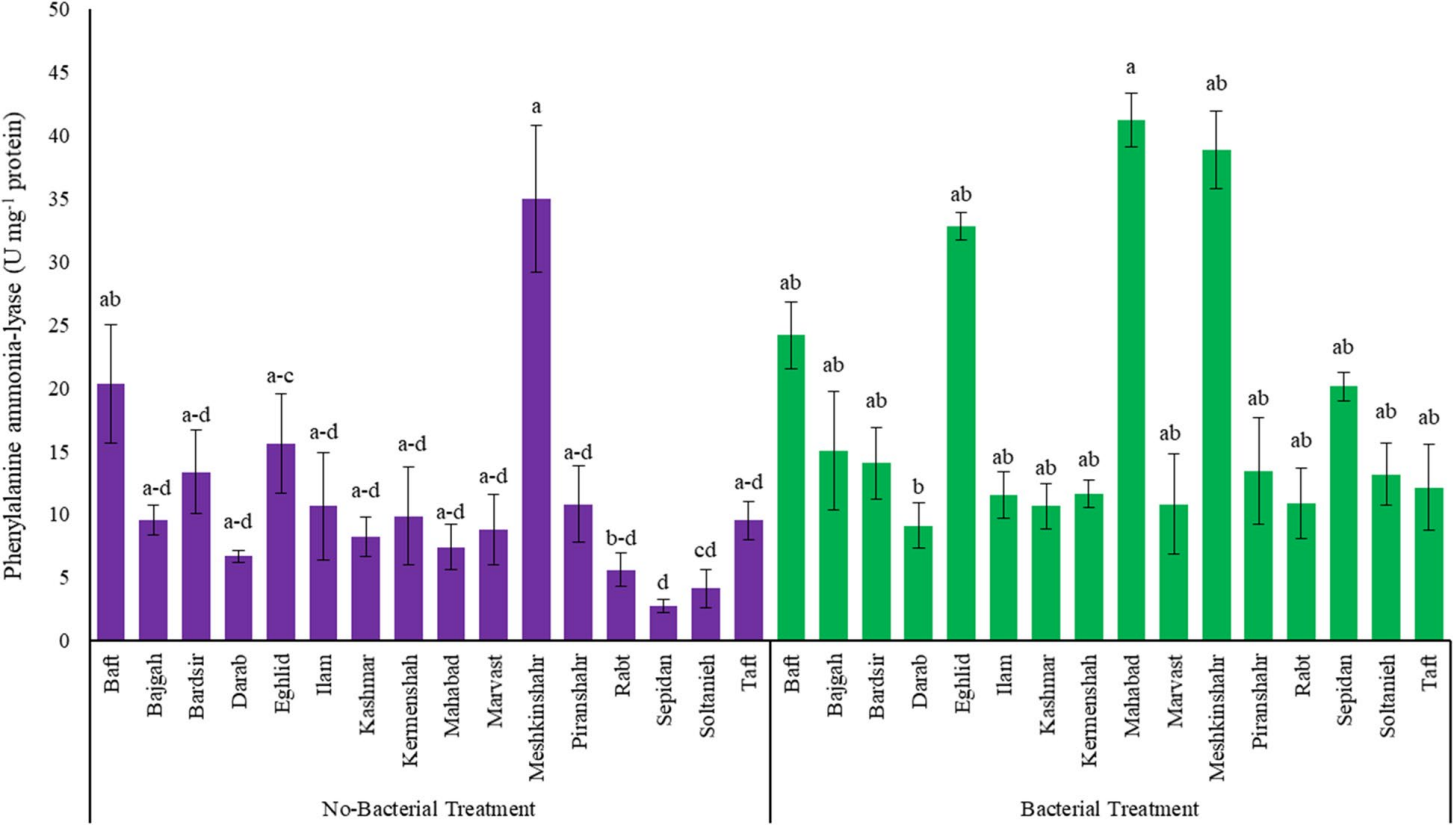
The Phenylalanine Ammonia-Lyase activity of Iranian Licorice accessions under Azotobacter application. According to the analysis of variance that double effects of Azotobacter and accession showed a significant difference, the slice method was used for mean comparisons. Columns with the same letter are not significantly different (*p* ≤ 0.05) (Tukey test). Bars stand for standard error (SE)

The present study results showed that the activity of enzymes in the plant antioxidant system increased significantly (*p* ≤ 0.05) under the interaction of the symbiotic bacteria and salinity (Table S1, Figs. 4 and 5). In this regard, the highest activities of PPO (0.21 U mg^−1^ protein), POD (3.09 U mg^−1^ protein U mg^−1^ protein), and PAL (17.85 U mg^−1^ protein) enzymes were observed (Fig. 4). However, under salinity stress, the activity of these enzymes significantly (*p* ≤ 0.05) decreased (Fig. 4). According to Fig. 5, the application of bacteria increased the activity of the PAL enzyme in all examined licorice accessions, with the most significant increase belonging to the accessions of Sepidan and Mahabad by 630.9 and 455.2%, respectively. Conversely, Bardsir and Ilam with a 5 and 7.8% increase in the activity of the PAL enzyme, showed the lowest percentage in increasing the activity of this enzyme (Fig. 5). The findings of different accessions of Iranian licorice to the interaction of bacteria and salinity are shown in Table S2. Interactions of accession × Azotobacter × salinity (G × A × S) for six biochemical parameters including CAT, SOD, POD, H_2_O_2_, MDA, and antioxidant activity were significant at the level of 5% probability. Then the analysis of mean comparisons was performed, which showed that in G × A_0_ × S0, G × A_0_ × S_1_, and G × A_1_ × S_0_ with the addition of salinity and Azotobacter, the activity of CAT, SOD, and POD enzymes in different accessions increased, and only in G × A_1_ × S_1_, the enzymatic activity of the accessions was decreased. Marvast and Piranshahr accessions irrigated with sodium chloride showed the highest increase in CAT enzyme (without bacteria), 281.31 and 186.59% increase, respectively. Also, in the presence of bacteria (with and without salinity stress), the highest level of CAT enzyme activity was observed in the Meshkinshahr accession (21.82 U mg^−1^ protein). The accession of Marvast (without bacteria) (2.53 U mg^−1^ protein) and Ilam (in the presence of bacteria) (2.78 U mg^−1^ protein) showed the highest activity in the SOD enzyme under salinity stress. Two accessions of Baft (without bacteria) (82.92 U mg^−1^ protein) and Sepidan (inoculated with bacteria) (94.34 U mg^−1^ protein) showed the highest enzymatic activity related to APX under salinity stress. In the present study, the bacteria were able to increase the level of SOD enzyme activity as the first defense against stress under salinity stress in many accessions, including Kermanshah, Taft, and Mahabad with 344, 240, and 119% increases, respectively.

### Principle component analysis (PCA), correlation, and cluster analysis

**Fig. 6:**
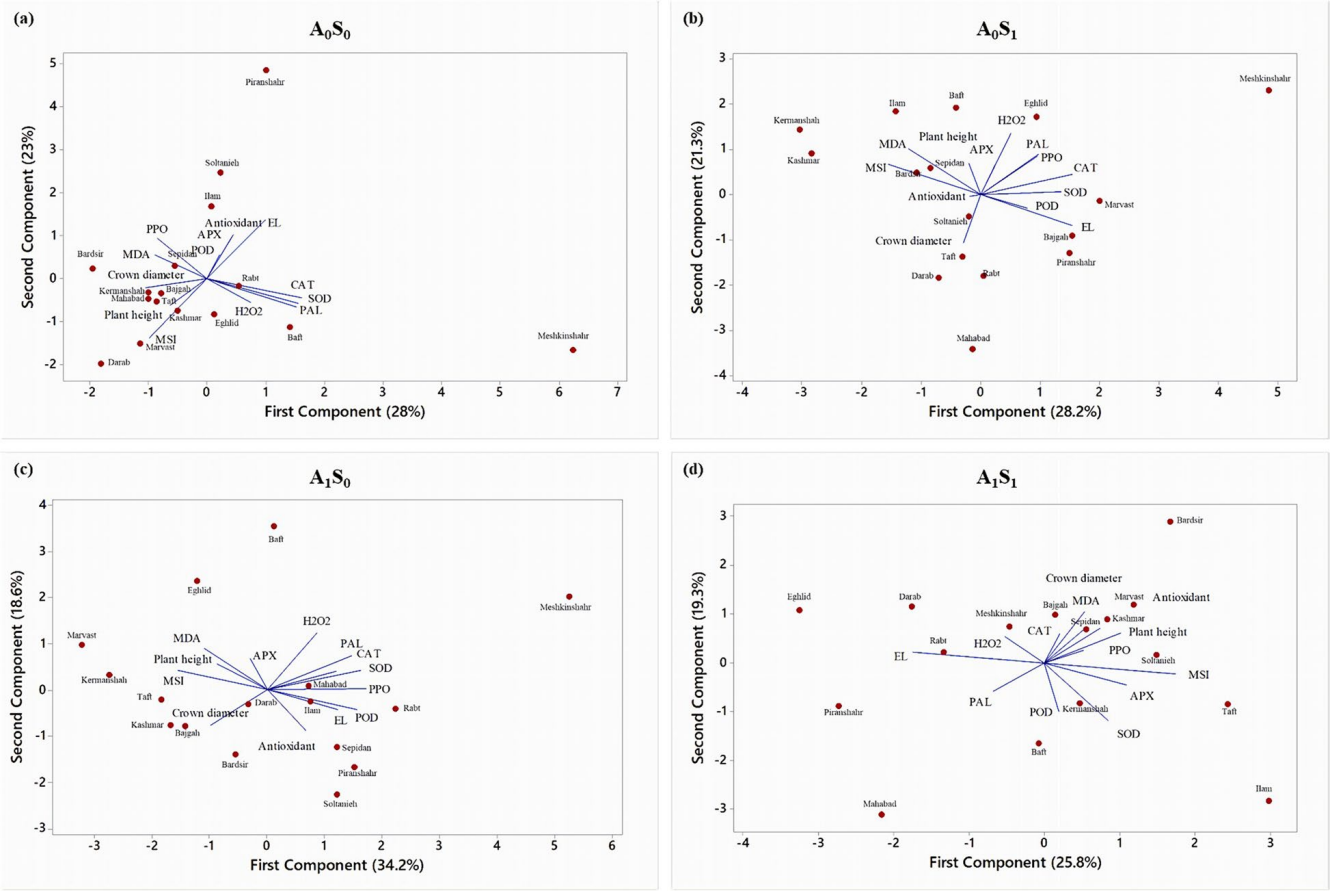
PCA-Biplot for the investigation of Iranian licorice accessions under studied treatments. **a** A_0_S_0_ (control); **b** A_0_S_1_ (no-bacterial treatment × salinity); **c** A_1_S_0_ (bacterial treatment × no-salinity); **d** A_1_S_1_ (bacterial treatment × salinity). CAT (Catalase), SOD (Superoxide dismutase), APX (Ascorbate peroxidase), POD (Peroxidase), PPO (Polyphenol oxidase), PAL (Phenylalanine Ammonia-Lyase), MDA (Malondialdehyde), EL (electrolyte leakage) and MSI (Membrane stability index)

**Fig. 7:**
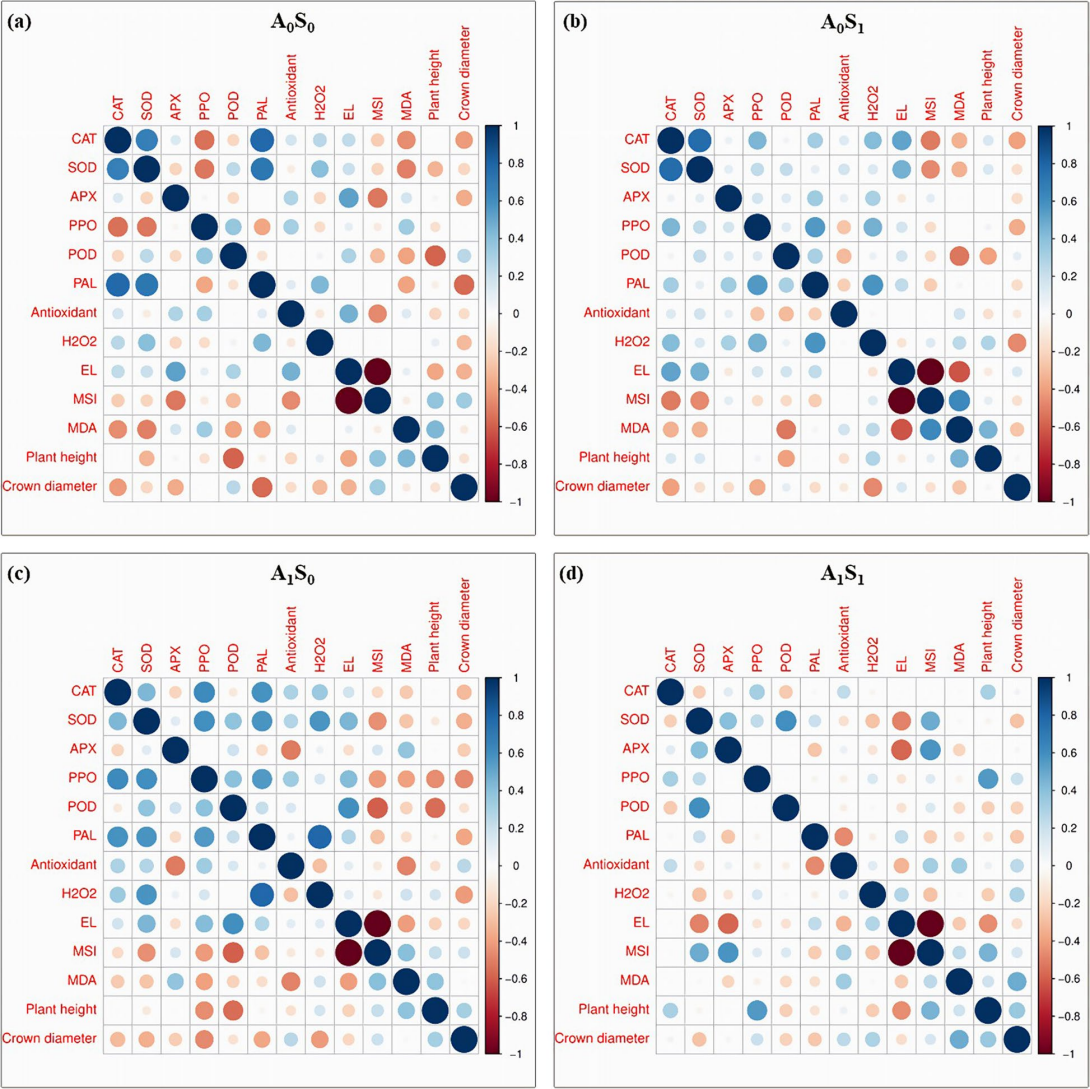
Corrplot between various measured parameters of Iranian licorice accessions under studied treatments. **a** A_0_S_0_ (control); **b** A_0_S_1_ (no-bacterial treatment × salinity); **c** A_1_S_0_ (bacterial treatment × no-salinity); **d** A_1_S_1_ (bacterial treatment × salinity). CAT (Catalase), SOD (Superoxide dismutase), APX (Ascorbate peroxidase), POD (Peroxidase), PPO (Polyphenol oxidase), PAL (Phenylalanine Ammonia-Lyase), MDA (Malondialdehyde), EL (electrolyte leakage) and MSI (Membrane stability index)

This study utilized the PCA and applied it to a matrix linking Iranian licorice accessions for individual identities to show a distinction between the Iranian licorice accessions and the morphophysiological and biochemical factors affecting the two variables. The graphs of separated treatments are shown in Fig. 6. A model of the distribution of Iranian licorice accessions based on complicated morphophysiological and biochemical variables was developed using principal component analysis. In the biplot of PCA analysis of control treatment (Fig. 6a), the first two factors accounted for 51% of the variability. In this plot, Meshkinshahr and Piranshahr were completely separate from other accessions. The plots of alone salinity treatment (Fig. 6b) and alone Azotobacter treatment (Fig. 6c) accounted for 49.5 and 52.8% of the total variance in both Meshkinshahr accession showed a separated performance. The last PCA shows integrated Azotobacter application and salinity stress (45.1% of total variance). According to almost all these analysis, it was found that the accession of Meshkinshahr showed a more remarkable ability to activate its enzymatic defense system under salt stress. Using correlation analysis, there were successful evaluations of the relations among licorice studied traits, including enzymes, morphophysiological traits, and biochemical traits (Figs. 7a, b, c, and d). Stronger positive correlations were found between H_2_O_2_, PAL, CAT, SOD, and PPO. Negative correlations Among PAL, plant height, MSI, EL, crown diameter, and POD were observed.

**Fig. 8:**
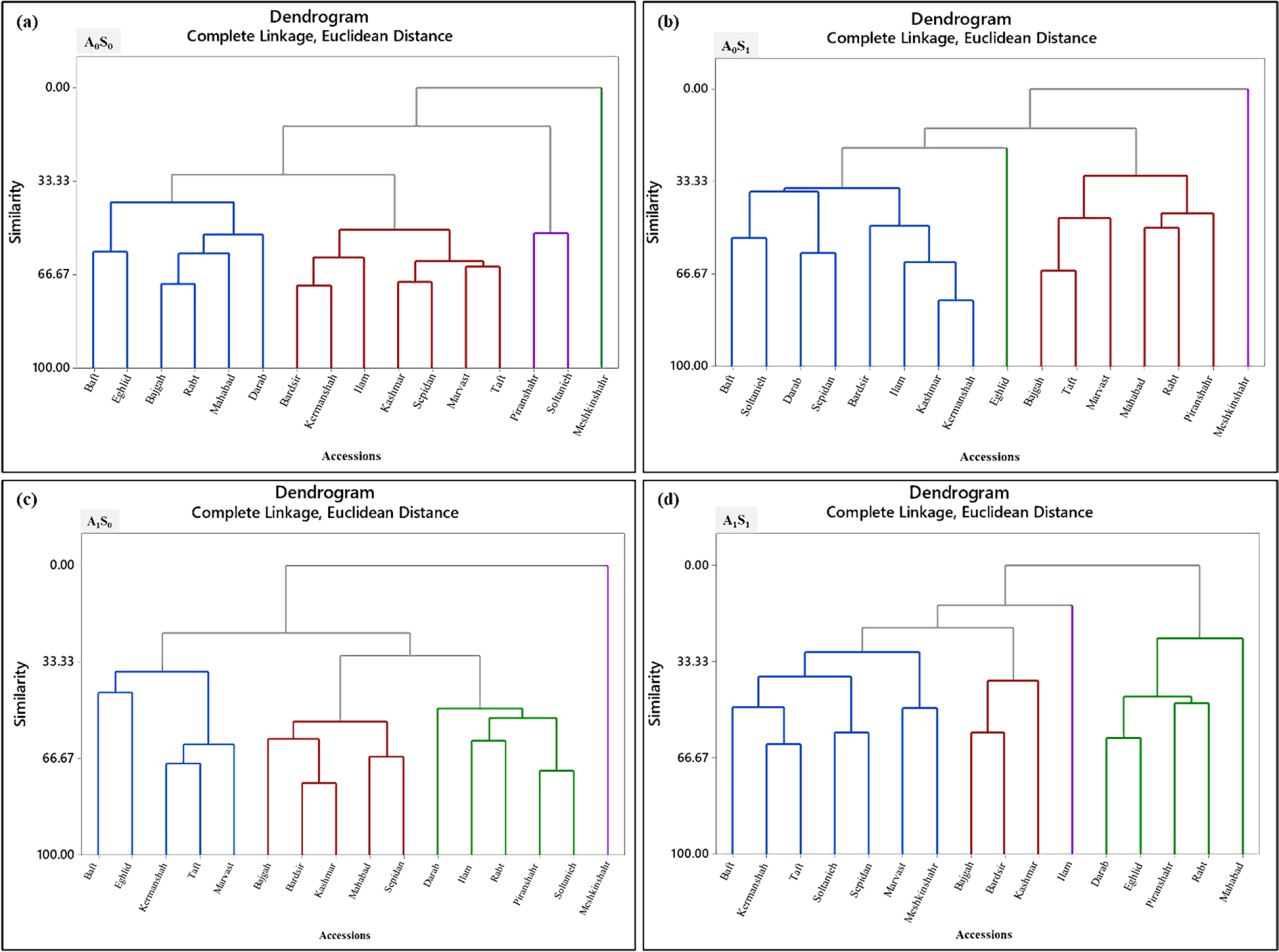
Cluster analysis of Iranian licorice accessions under studied treatments. **a** A_0_S_0_ (control); **b** A_0_S_1_ (no-bacterial treatment × salinity); **c** A_1_S_0_ (bacterial treatment × no-salinity); **d** A_1_S_1_ (bacterial treatment × salinity)

The cluster analysis was done based on complete linkage and Euclidean distance into four separated groups (Figs. 8a, b, c, and d). Three accessions of Meshkinshahr, Eghlid, and Ilam were categorized in separate clusters than other accessions that were almost similar to the results of PCA analysis.

## Discussion

### Morphophysiological parameters

#### Crown diameter and plant height

When plants are exposed to environmental stresses, the equilibrium between ROS generation and their detoxification by the antioxidative system can be disrupted, resulting in oxidative damage [45]. Similar to the present research results, various accessions have different levels of tolerance to these environmental stresses, which is linked to their ability to scavenge ROS [45, 46]. The responses of some accessions in the present study are in line with the above. Salinity decreased the plant growth and membrane stability index in a tomato plant [47]. Other studies have found a reduction in stem diameter in pistachio accessions under salt stress [48], similar to our findings. According to a previous study, the primary explanations for the reduction in stem diameter of pistachio seedlings were the limits of photo-assimilates accumulation and root function in water transport under salt stress [49]. A study on rice cultivars showed that plant height cultivars were significantly (*p* ≤ 0.05) reduced by salinity. Because the hydrolysis of stored nutrients and their transfer to the developing shoots is inhibited by salt, the xylem's growth is slowed. Screening and identifying salt-tolerant varieties, genotypes, or accessions can be a useful management approach for reducing salinity effects and increasing agricultural production [50]. Other research on cowpea, (*Vigna unguiculata* L.), and *Brassica campestris* L. also found that lower salt levels (< 4dsm^−1^) increased plant height, whereas higher salinity levels (> 4dsm^−1^) resulted in a decrease in plant height [51, 52]. Many plants have shown improved salinity stress tolerance and increased plant growth after PGPB inoculation, including [53], cotton [54], oat [55], chickpea [56], chili pepper [57], licorice [27], mung beans [58], and mint [59]. Rhizospheric microorganisms are associated with the development of root systems by dissolving high-energy elements such as P or K, increasing the ability of plants to absorb elements from the soil [60].

### Electrolyte leakage and leaf membrane stability index

Cell membrane stability has long been used to distinguish stress-tolerant from stress-susceptible cultivars, and greater membrane stability has been linked to abiotic stress tolerance [61]. Similar to the results of the present study, the effect of salinity treatment on the electrolyte leakage rate of three *Lactuca sativa* L., *Tetragonia tetragonoides* L., and *Portulaca oleracea* L. plants, resulted in salinity levels increased, the amount of electrolyte leakage increased in all the three plants [62]. MSI reduced under salt stress for two weeks following salinity, according to Ansari et al. (2019), and MSI was greater in inoculation plants than in non-inoculated plants. In a study on *Medicago sativa* L. at 20 dS m^− 1^, inoculated plants of two examined accessions, Heris and Hashtrod, exhibited a greater MSI than others, but at 10 dS m^− 1^ a week after salinity treatment, there was no notable variation in MSI between inoculated and non-inoculated treatments [63]. Variations in the cultivar responses to salinity may indicate that their genetic value is adaptive [64]. Under salinity stress, a lack of water and a high amount of ROS might cause lower MSI [65, 66]. Because of reduced Na^+^ ion absorption and a greater RWC, MSI in inoculated plants was more than in non-inoculated ones [63]. Similarly, due to water and nutrient absorption enhancement, researchers stated that inoculated seedlings represented fewer signs of oxidative destruction and high membrane stability under salinity [67]. Werner and Newton (2005) discovered that inoculated plants displayed reduced oxidative destruction and high membrane stability during salt stress due to enhanced water and nutrient uptake [67]. PGPRs cause up-regulation of stress tolerance genes such as RAB18 (LEA), the RD29A and RD29B regulons of ABA-responsive elements (ABRE), and dehydration responsive elements (DRE), as well as the transcription factor DREB2b DRE binding protein [68].

### H_2_O_2_, MDA, and antioxidant activity assay

In this study, the results of hydrogen peroxide, malondialdehyde, and antioxidant activity measurements were consistent with the results of some researchers worldwide, which will be mentioned. The presence of H_2_O_2_ is often used as a marker for oxidative stress. H_2_O_2_ is harmful to cells and must be detoxified to water and oxygen by CAT and/or POD [69, 70]. Active oxygen species, which are powerful oxidizers, are generated under stress and can harm a variety of cellular components, including carbohydrates, proteins, lipids, and nucleic acids, finally leading to cell death [69, 71]. The H_2_O_2_ concentration of black *Arthrocnemum macrostachyum* L. seeds was greater than brown seeds and increased with increases in salinity. At 400 mM NaCl, the H_2_O_2_ content of tiny seeds of *A. Indicum* enhances, but the concentration of big seeds was unaffected [72]. Antioxidative enzymes generated by the PGPR are thought to contribute to plant salt stress tolerance by removing H_2_O_2_ from salinity-stressed roots [73]. Under salt stress, MDA, a result of the breakdown of polyunsaturated fatty acids in biomembranes, accumulates more. When plants are subjected to salinity, MDA is deposited in tissues to indicate membrane destruction [66]. Similar to the present results, an increased degree of lipid peroxidation was detected in the chloroplast of tomato seedlings subjected to salt stress, as shown by MDA content and H_2_O_2_ content, indicating that salt stress caused oxidative damage chloroplast of tomato seedlings. To deal with the oxidative stress, the antioxidant capacity of tomato seedling chloroplasts was increased [74]. A reduction in MDA levels in salt-treated rice plantlets under *Enterobacter* sp. P23 application was observed [75]. Similarly, *Pseudomonas pseudoalcaligenes* reduce lipid peroxidation by promoting salinity-affected rice GJ-17 [76]. PGPRs activate plant antioxidant defense machinery by upregulating the activity of key enzymes that scavenge excess ROS [16]. Singh and Tiwari stated that under saline situations, MDA content in the leaves had a rectilinear enhancement with enhancing salinity levels in microbial inoculated and uninoculated HD 2967 wheat cultivar. At each level of salinity, PGPB and *Piriformospora indica* L. inoculated plants had lower levels of MDA than non-inoculated control plants. They also stated that at 0 and 200 mM NaCl treatments, there was no notable difference in lipid peroxidation levels between PGPB and *P. indica* [77]. Salt stress causes a significant rise in MDA concentration, implying that under salinity membrane lipid degradation may accelerate, resulting in increased membrane permeability, electrolyte exosmosis, and, eventually, damage to cell membrane systems [78]. The reduced MDA concentration suggests that seedlings inoculated with *B. aquimaris* DY-3 are more resistant to stress under salt conditions, which is supported by the lower Na^+^ quantity and suggested that decreased the ion toxicity caused by Na^+^ [79]. Plants have a sophisticated system that is hierarchically organized and made up of interacting components that can inhibit free radical production. For continuous hydrogen abstraction and free radical scavenging, plants maintain complex networks of overlapping antioxidants [80]. In a study on two species of *Thymus*, Bistgani et al. (2019) discovered that salt stress enhances the antioxidant activity of this plant. The extracts derived from plants treated with 90 mM NaCl had the highest antioxidant activity. *T. vulgaris* methanolic extract had the strongest antiradical activity (69.7% inhibition), which was slightly greater than *T. daenensis* (61.2% inhibition) following irrigation with 90 mM NaCl [81]. By examining the antioxidant activity of *Stevia rebaudiana* Bertoni. under salinity treatment and the use of growth-promoting microorganisms, Forouzi et al. (2020) found that inoculated plants with *Piriformospora indica* under a salinity level of 3 dS/m had the highest inhibitory activity, towards control. Increasing salinity enhanced free radical inhibitory activity in inoculated plants. When comparing the antioxidant efficacy of stevia leaf extract treated with fungus against *Streptomyces* isolates, it seems that plants treated with fungi have greater potency. A higher inhibitory activity can be attributed to phenylpropanoid content [82]. Alleviation of salt stress in *G. glabra* by arbuscular mycorrhiza is also reported previously. They observed that salinity caused a significant increase in electrolyte leakage and Na + concentration in the shoot and root of licorice [83].

### Enzyme assay

Evaluating the activity of different enzymes in Iranian licorice accessions showed that the present study results were consistent with some previous studies and contradicted some. Long-term saltiness reduces the activity of ROS-scavenging enzymes, including CAT, SOD, and APX, and promotes lipid peroxidation [84]. PGPRs activate antioxidant enzymes including SOD, and CAT to detoxify ROS, and protect the plants from salt toxicity Under salt stress, different antioxidant enzymes' activity has been observed to change. SOD is the leading free radical scavenger in plant cells. The main antioxidant enzymes that scavenge harmful H_2_O_2_ in the cytosol and chloroplasts of plant cells are POD and CAT [85]. Under saline conditions, PGPB treatment increased POD activity in plants. Salinity stress enhanced POD activity in chickpea plants significantly(*p* ≤ 0.05), and the level of POD was much higher in plants inoculated with *Bacillus subtilis* BERA 71 [86]. According to research on melon genotypes exposed to salt stress, enzyme activity reduced in response to the salt application, however, the responses differed among genotypes, particularly APX and SOD activity [87]. In a study conducted by Stassinos et al. on different cultivars of *Brassica napus*, it was discovered that the level of CAT enzyme activity was increased when exposed to mild salinity and inoculated with Arthrobacter globiformis increased significantly (*p* ≤ 0.05). This increase in PAL activity was linked to higher levels of phenolic compounds in inoculated samples (Stassinos et al., 2020). In chickpea plants micro-injected with *Pseudomonas aeruginosa*, Basha et al. (2006) found an increase in PAL activity and phenolic chemicals [88]. It can be concluded that the bacteria contributed to the plant enzyme system. Similar observations were achieved in the current study regarding enzymatic defence. PGPB has been shown in numerous studies to activate several genes in plants that encode antioxidant enzymes [89]. Most aerobic bacteria found in plants are catalase-positive and can help plants in times of stress by scavenging H_2_O_2_. After inoculation with the *Pseudomonas frederiksbergensis* strain, certain plants growing under high salt circumstances showed a reduction in APX and SOD enzyme activity levels. This drop-down may be explained as enzymes are engaged in the neutralization of free radicals, thus the amount of free enzymes is reduced, resulting in a decrease in enzyme synthesis, and they also promote plant development [90]. PGPR's molecular approach to plants is multi-directional and mutualistic. Bacterization of salinity-affected plants with PGPB reduces stress by boosting the activity of ROS-scavenging enzymes. An enhancement in POD activity of wheat under 5% salinity when inoculated with *P. fluorescens* Ms-01 and *P. fluorescens* Ms-01 + *A. brasilense* DSM1690 compared with non-inoculated seedlings showed a role of PGPB in preserving herbs in case of salt stress stimulated oxidative damage [91]. Similar to the present observations in various licorice accessions, plants differ greatly in their tolerance of salinity, as reflected in their different growth responses [92]. Supplementing salt-affected plants with PGPB increases defense enzymes, allowing plants to be resistant to salinity [93]. Moreover, an enhancement in CAT and POD activity was stated by Islam et al. (2016) when 9 dS m^−1^ salt-stressed *Vigna radiata* were inoculated by *Bacillus cereus* Pb25 [94]. Similar differences in antioxidant enzyme activity of salt-stressed plants and bacterized salt-stressed plants were stated by [95] for lettuce seedlings inoculated by *Pseudomonas mendocin*, *Solanum tuberosum* inoculated by *Bacillus* strains [90], and tomato seedlings with *P. extremorientalis* TSAU20 [96]. Various responses to salinity and Azotobacter inoculation in different accessions of licorice in the present study can because of inter-population variations, the genetic make-up of accessions, genetic factors, physiological variations, and evolution [97]. The PGPB influence on antioxidant enzyme-producing genes may explain the increase in ROS scavenging enzyme activity in salt-stressed plants following PGPB inoculation. Different reactions to PGPB inoculation in different accessions might be due to a distinct bacterium habitat on the root and rhizome of individual accessions. It was proposed that rhizobacteria's varied reactions plant root architecture revealed the uniqueness of plant-rhizobacterium interactions [54, 98, 99].

## Conclusion

The usage of Azotobacter appears to increase the enzymatic defense of Iranian licorice plants cultivated in saline soil, based on present research findings. Significant improvements in most morphophysiological and biochemical parameters were accession-specific. Malondialdehyde and H_2_O_2_ levels were inhibited after Azotobacter inoculation with specific accessions, indicating reduced salt-stress toxicity. Enzymatic activities (PPO, SOD, PAL, CAT, APX) were promoted in Meshkinshahr, Eghlid, Baft, and Ilam for the Azotobacter inoculation, as a result, plants' resistance to salt stress is increased. So, these mentioned genotypes can be introduced as more tolerant ones, according to the measured parameters. This study suggested that using inoculation with Azotobacter and accession selection could be a helpful and crucial method to relieve salt stress.

## Supplementary Information

**Additional file 1: Table S1.** Effect of Azotobacter and salinity stress interaction on studied biochemical parameters of Iranian licorice accessions. **Table S2.** Effect of Azotobacter and salinity interaction on the enzyme activities of different Iranian licorice accessions. **Table S3.** Analyze variance of Irainin licorice accessions biochemical traits understudied treatments. **Table S4.** Analyze variance of Irainin licorice accessions biochemical traits understudied treatments. **Table S5.** Analyze variance of Irainin licorice accessions biochemical traits understudied treatments.

## Data Availability

The dataset generated during and/or analyzed during the current study is available from the corresponding author upon reasonable request.

## Abbreviations

A_0_: Non-inoculated by Azotobacter
A_1_: Inoculated by Azotobacter
NaCl: Sodium chloride
S_0_: Without salinity stress
S_1_: Under salinity stress
MSI: Membrane stability index
PPO: Polyphenol oxidase
POD: Peroxidase
PAL: Phenylalanine Ammonia-Lyase
PTCC: Persian Type Culture Collection
PCA: Principal Component Analysis
Na^+^: Sodium
Cl: Chloride
ROS: Reactive oxygen species
PGPB: Plant growth-promoting bacteria
PGPR: Plant growth-promoting rhizobacteria
CAT: Catalase
SOD: Superoxide dismutase
APX: Ascorbate peroxidase
EL: Electrolyte leakage
MDA: Malondialdehyde
PVP: Polyvinylpyrrolidone
NBT: Nitroblue-tetrazolium;
DPPH: 2, 2-Diphenyl-1-picrylhydrazyl;
EC: Electrical conductivity
TCA: Trichloroacetic acid
TBA: Thiobarbituric acid
KI: Potassium iodide
CRD: Completely randomized design
ANOVA: Analysis of variance
SE: Standard error
