# Supplementary material for: Microbial amelioration of salinity stress in endangered accessions of Iranian licorice (Glycyrrhiza glabra L.)
Source: BMC Plant Biol. 2022 Jul 5;22:322. doi: 10.1186/s12870-022-03703-9 (PMC9254424; doi:10.1186/s12870-022-03703-9)
Supplement: Supplementary file 1 — Additional file 1: Table S1. Effect of Azotobacter and salinity stress interaction on studied biochemical parameters of Iranian licorice accessions. Table S2. Effect of Azotobacter and salinity interaction on the enzyme activities of different Iranian licorice accessions. Table S3. Analyze variance of Irainin licorice accessions biochemical traits understudied treatments. Table S4. Analyze variance of Irainin licorice accessions biochemical traits understudied treatments. Table S5. Analyze variance of Irainin licorice accessions biochemical traits understudied treatments. [file 12870_2022_3703_MOESM1_ESM.docx]

Table S1. Effect of Azotobacter and salinity stress interaction on studied biochemical parameters of Iranian licorice accessions.

|  | Traits | | | | | | | | | | | | | | | | |
| --- | --- | --- | --- | --- | --- | --- | --- | --- | --- | --- | --- | --- | --- | --- | --- | --- | --- |
| No. | Accessions | Hydrogen peroxide  (nmol/g FW) | | | | | | | Malondialdehyde  (µmol/g FW) | | | | | Antioxidant Activity  (%) | | | |
|  |  | A_0_ | | | | A_1_ | | | | A_0_ | | A_1_ | | A_0_ | | A_1_ | |
|  |  | S_0_ | S_1_ | | S_0_ | | S_1_ | | | S_0_ | S_1_ | S_0_ | S_1_ | S_0_ | S_1_ | S_0_ | S_1_ |
| 1 | Baft | 15^b^±1.35 | | 22^ab^±1.18 | | 23^a^±0.5 | | 7^de^±0.25 | 21.46^ab^±2.31 | | 25.73^ab^±7.44 | 28.93^ab^±8.38 | 16.55^b-d^±4.16 | 73.49^a-c^±1.41 | 74.00^a-d^± 0.69 | 69.53^b-d^±0.17 | 67.68^b^±0.61 |
| 2 | Bajgah | 6^ef^±0.86 | | 6^de^±0.04 | | 3^de^±0.34 | | 8^de^±0.73 | 13.52^a-c^±2.97 | | 17.68^bc^±1.00 | 19.44^ab^±4.22 | 23.5^a-c^±3.4 | 70.68^b-d^± 0.64 | 74.12^a-d^±1.49 | 76.52^a^±0.6 | 75.42^a^±945 |
| 3 | Bardsir | 4^ef^±0.48 | | 17^a-c^±2.28 | | 3^de^±0.07 | | 16^bc^±0.55 | 35.43^a^±4.17 | | 38.49^a^±5.62 | 12.38^ab^±5.12 | 41.71^ab^±2.18 | 72.12^a-d^±2.59 | 73.49^a-d^±1.22 | 73.37^a-d^±1.35 | 76.28^a^±0.317 |
| 4 | Darab | 4^ef^±0.21 | | 5^e^±0.86 | | 8^b-d^±0.68 | | 11^cd^±0.18 | 7.91^bc^±3.38 | | 19.57^ab^±3.34 | 18.85^ab^±2.7 | 21.29^bc^±8.2 | 66.65^d^±0.13 | 69.64^d^±0.7 | 68.5^d^±0.55 | 72.92^ab^±1.3 |
| 5 | Eghlid | 22^a^±0.87 | | 23^ab^±0.42 | | 7^c-e^±0.38 | | 22^a^±0.11 | 23.01^ab^±6.03 | | 27.93^ab^±7.91 | 29.58^a^±3.56 | 22.43^a-c^±2.5 | 69.13^b-d^±0.05 | 71.1^cd^±0.07 | 68.73^cd^±0.66 | 71.6^ab^±1.29 |
| 6 | Ilam | 4^ef^±0.1 | | 17^a-c^±1.74 | | 11^bc^±0.39 | | 12^cd^±0.12 | 23.52^ab^±0.74 | | 37.26^a^±1.1 | 16.34^ab^±2.3 | 24.25^a-c^±9.55 | 74.98^ab^±1.69 | 75.64^a-c^±0.94 | 74.75^ab^±0.94 | 75.27^a^±2.03 |
| 7 | Kashmar | 4^c-e^±1.54 | | 7^de^±1.24 | | 5^c-e^±0.44 | | 4^e^±0.23 | 20.3^ab^±10.4 | | 40.45^a^±7.13 | 11.67^b^±2.39 | 50.34^a^±3.1 | 69.42^b-d^±0.1 | 75.3^a-c^±0.65 | 74.06^a-c^±1.84 | 76.23^a^±0.1 |
| 8 | Kermanshah | 8^ef^±0.48 | | 14^c^±2.48 | | 6^c-e^±0.19 | | 3^e^±0.12 | 21.12^ab^±2.25 | | 41.1^a^±10.8 | 21.6^ab^±9.3 | 12.25^cd^±4.76 | 74.92^a-b^±0.63 | 76.35^ab^±0.45 | 74.58^ab^±0.9 | 75.59^a^±0.167 |
| 9 | Mahabad | 3^f^±0.12 | | 5^e^±1.23 | | 13^b^±0.43 | | 3^e^±0.1 | 10.8^b-c^±1.33 | | 11.52^b^±1.91 | 16.41^ab^±3.13 | 15.65^cd^±2.76 | 74.81^a-b^±2.18 | 75.87^a-c^±0.27 | 74.6^ab^±0.5 | 67.29^b^±0.005 |
| 10 | Marvast | 12^bc^±0.37 | | 16^bc^±2.59 | | 7^c-e^±0.89 | | 4^e^±0.56 | 17.27^a-c^±1.24 | | 25.00^ab^±4.07 | 24.05^ab^±4.66 | 17.40^bc^±0.97 | 69.38^b-d^±0.49 | 74.41^a-d^±2.24 | 74.18^a-c^±1.82 | 71.77^ab^±1.43 |
| 11 | Meshkinshahr | 13^bc^±1.23 | | 24^a^±1.28 | | 19^a^±0.39 | | 13^cd^±0.14 | 2.4^c^±0.182 | | 18.85^ab^±3.22 | 15.72^ab^±0.67 | 21.19^bc^±3.73 | 73.03^a-c^±1.1 | 74.35^a-d^±1.49 | 76.01^a^±0.77 | 75.67^a^±0.639 |
| 12 | Piranshahr | 3^f^±0.28 | | 5^e^±0.57 | | 4^d-e^±0.16 | | 15^b-c^±0.98 | 18.37^a-c^±6.59 | | 23.17^ab^±4.59 | 13.5^ab^±0.74 | 2.48^d^±0.23 | 74.98^ab^±1.24 | 76.64^a-b^±0.45 | 75.96^a^±1.21 | 75.00^a^±0.774 |
| 13 | Rabt | 6^ef^±0.3 | | 6^e^±0.02 | | 7^c-e^±0.3 | | 4^e^±0.16 | 15.3^a-c^±4.75 | | 17.65^ab^±3.89 | 15.1^ab^±4.21 | 26.98^a-c^±3.7 | 68.45^cd^±0.59 | 72.51^b-d^±0.35 | 73.08^a-d^±0.84 | 75.21^a^±1.29 |
| 14 | Sepidan | 5^ef^±0.62 | | 13^cd^±0.81 | | 7^c-e^±0.83 | | 19^ab^±0.18 | 24.84^ab^±4.12 | | 24.63^ab^±2.47 | 13.97^ab^±0.65 | 16.37^b-d^±3.74 | 70.22^b-d^±0.65 | 71.94^b-d^±0.29 | 76.23^a^±0.56 | 74.18^a^±1.07 |
| 15 | Soltanieh | 10.5^cd^±0.18 | | 17^a-c^±1.28 | | 2^e^±0.01 | | 8^de^±0.4 | 19.44^ab^±1.66 | | 21.67^ab^±2.35 | 17.23^ab^±3.47 | 14.93^cd^±4.36 | 77.29^a^±0.4 | 78.03^a^±0.01 | 76.92^a^±0.48 | 74.95^a^±0.944 |
| 16 | Taft | 4^f^±0.31 | | 5^e^±0.44 | | 7^b-e^±0.15 | | 4^e^±0.04 | 15.06^a-c^±3.62 | | 26.79^ab^±6.95 | 24.05^ab^±2.62 | 19.22^bc^±2.74 | 72.74^a-d^±1.38 | 75.21^a-c^±0.97 | 72.8^a-d^±1.54 | 74.93^a^±1.95 |

S stands for salinity (S_0_; no salinity application, and S_1_; 200 mM NaCl,) and A standsd for Azotobacter inoculation (A_0_; non-inoculation, and A_1_; inoculation by Azotobacter). Mean values with the same superscript within a column are not significantly different (*p* ≤ 0.05). Tukey test. Means± Standard error (SE).

| Traits | | | | | | | | | | | | | | | | | | | | | | | | | | | |
| --- | --- | --- | --- | --- | --- | --- | --- | --- | --- | --- | --- | --- | --- | --- | --- | --- | --- | --- | --- | --- | --- | --- | --- | --- | --- | --- | --- |
| No. | Accessions | Catalase  (U mg^-1^ protein) | | | | | | | | Superoxide dismutase  (U mg^-1^ protein) | | | | | | | Ascorbate peroxidase  (U mg^-1^ protein) | | | | | | | | | | |
|  |  | A_0_ | | | A_1_ | | | A_0_ | | | | | A_1_ | | | | | A_0_ | | | | A_1_ | | | | |  |
|  |  | S_0_ | S_1_ | S_0_ | | S_1_ | | | S_0_ | | S_1_ | | S_0_ | | S_1_ | | | | S_0_ | S_1_ | | | S_0_ | | S_1_ | | |
| 1 | Baft | 5.31^b-e^± 1.93 | 9.45^b-c^ ± 2.19 | 5.63^b-e^ ± 0.21 | | | 2.93^g^ ± 0.26 | | 1.11^ab^ ± 0.07 | | | 1.28^ab^±0.03 | | 1.11^a-d^ ±0.2 | | 1.08^bc^ ±0.032 | | | 16^i^ ±0.56 | | 82.92^a^ ±0.68 | | | 56.28^a-c^ ±1.71 | | 48.91^c-e^ ±0.35 | |
| 2 | Bajgah | 3.81^c-e^± 0.56 | 8.96^bc^ ± 1.04 | 6.26^b-e^ ± 0.15 | | | 5.98^c-g^ ± 0.48 | | 0.69^ab^ ±0.28 | | | 1.38^ab^±0.57 | | 0.8^a-d^ ±0.276 | | 0.70^b-d^ ±0.141 | | | 23.19^f-h^ ±2.09 | | 74.7^ab^±1 | | | 19.35^fg^ ±0.7 | | 36.98^e-g^ ±1.54 | |
| 3 | Bardsir | 4.7^b-e^ ± 0.64 | 5.34^c-e^ ± 0.39 | 10.05^b^ ± 0.33 | | | 6.75^b-f^ ± 0.24 | | 0.26^b^ ±0.06 | | | 0.48^b^±0.03 | | 0.21^d^ ±0.024 | | 0.42^cd^ ±0.114 | | | 31.18^b-f^ ±0.85 | | 38.67^e-g^ ±2.18 | | | 37^c-e^ ±2.77 | | 34.17^f-h^ ±4.04 | |
| 4 | Darab | 2.73^e^± 0.19 | 4.37^de^ ± 0.65 | 3.03^f^ ± 0.45 | | | 5.71^d-g^ ± 0.55 | | 0.43^ab^ ±0.18 | | | 0.85^ab^±0.44 | | 0.39^c-d^ ±0.145 | | 0.35^cd^ ±0.160 | | | 16.53^hi^ ±0.49 | | 49.2^c-g^ ±4.74 | | | 28.39^d-f^ ±4.86 | | 18.66^j^ ±2.39 | |
| 5 | Eghlid | 4.80^b-e^± 0.46 | 4.95^bc^ ± 0.09 | 9.3^b^ ± 0.24 | | | 8.89^a-e^ ± 0.47 | | 0.63^ab^ ±0.19 | | | 0.64^ab^±0.29 | | 0.41^c-d^ ±0.115 | | 0.35^cd^ ±0.041 | | | 27.76^c-g^±0.35 | | 59.84^a-d^ ±0.39 | | | 83.21^a^ ±0.47 | | 20.22^ij^ ±2.19 | |
| 6 | Ilam | 5.82^b-d^ ± 0.55 | 7.85^b-d^ ± 0.29 | 6.38^b-e^ ± 0.79 | | | 3.84^fg^ ± 1.1 | | 0.37^ab^ ±0.04 | | | 1.23^ab^±0.37 | | 1.74^ab^ ±0.066 | | 2.78^a^ ±0.325 | | | 38.84^ab^ ±1.31 | | 46.24^c-g^ ±0.63 | | | 54.08^a-c^ ±0.91 | | 74.45^ab^ ±2.68 | |
| 7 | Kashmar | 3.66^c-e^ ± 0.14 | 3.88^e^ ± 0.38 | 8.69^bc^ ± 0.86 | | | 4.75^e-g^ ± 1.29 | | 0.75^ab^ ±0.27 | | | 0.86^ab^±0.44 | | 0.39^c-d^ ±0.115 | | 0.62^b-d^ ±0.319 | | | 34.88^bc^±0.79 | | 48.89^c-g^ ±0.45 | | | 33.21^c-f^ ±3.33 | | 28.03^g-I^ ±1.56 | |
| 8 | Kermanshah | 4.99^b-e^± 0.27 | 6.35^c-e^ ± 1.18 | 4.81^c-f^ ± 0.44 | | | 3.30^fg^ ± 0.29 | | 0.37^ab^ ±0.12 | | | 0.52^ab^±0.18 | | 0.25^d^ ±0.001 | | 1.11^bc^ ±0.287 | | | 33.49^b-d^±0.8 | | 54.45^b-f^ ±0.76 | | | 43.98^b-d^ ±1.21 | | 53.58^b-d^ ±0.8 | |
| 9 | Mahabad | 3.93^b-e^ ± 0.6 | 7.63^b-e^ ± 1.15 | 6.99^b-d^ ± 1.87 | | | 3.81^fg^ ± 0.38 | | 0.58^ab^ ±0.04 | | | 0.69^ab^±0.06 | | 0.72^a-d^ ±0.172 | | 1.58^ab^ ±0.164 | | | 20.51^g-i^ ±2.96 | | 23.41^h^ ±3.74 | | | 29.97^d-f^ ±8.72 | | 42.5^d-f^ ±4.05 | |
| 10 | Marvast | 3.8^c-e^ ± 0.39 | 14.49^ab^ ± 0.45 | 5.41^b-f^ ± 0.23 | | | 13.90^ab^± 2.17 | | 0.49^ab^ ±0.22 | | | 2.53^a^±0.45 | | 0.42^c-d^ ±0.214 | | 0.62^b-d^ ±0.047 | | | 33.15^b-e^±1.14 | | 34.56^g^ ±1.11 | | | 53.29^a-c^ ±1.63 | | 47.95^c-e^ ±1.26 | |
| 11 | Meshkinshahr | 17.84^a^ ± 3.1 | 25.04^a^ ± 0.43 | 21.82^a^ ± 0.51 | | | 17.68^a^ ± 1.38 | | 1.32^a^±0.62 | | | 2.08^ab^±0.72 | | 1.96^a^ ±0.518 | | 0.9^b-d^ ±0.356 | | | 34.63^bc^ ±0.67 | | 57.1^b-f^ ±13.1 | | | 21.79^ef^ ±2.11 | | 26.07^h-j^ ±2.59 | |
| 12 | Piranshahr | 2.91^de^ ± 0.18 | 8.34^b-d^ ± 0.53 | 4.02^d-f^ ± 0.72 | | | 4.36^e-g^ ± 0.51 | | 0.51^ab^ ±0.15 | | | 1.51^ab^±0.45 | | 0.83^a-d^ ±0.262 | | 0.17^d^ ±0.008 | | | 50.16^a^ ±2.94 | | 57.18^a-e^ ±1.18 | | | 37.27^c-e^ ±3.71 | | 20.65^ij^ ±0.67 | |
| 13 | Rabt | 6.78^b-c^ ± 0.64 | 7.59^c-e^ ± 1.86 | 3.41^ef^ ± 0.19 | | | 12.05^a-c^ ± 0.78 | | 0.94^ab^ ±0.13 | | | 1.24^ab^±0.07 | | 1.39^a-c^ ±0.205 | | 0.6^b-d^ ±0.116 | | | 24.78^d-g^ ±1.71 | | 36.78^fg^ ±0.96 | | | 74.28^ab^ ±1.95 | | 54.7^b-d^ ±0.44 | |
| 14 | Sepidan | 4.38^b-e^ ± 0.59 | 7.66^b-e^ ± 0.26 | 7.66^bc^ ± 0.1 | | | 5.62^d-g^ ± 0.46 | | 0.65^ab^ ±0.03 | | | 0.91^ab^±0.21 | | 0.56^b-d^ ±0.187 | | 0.52^b-d^ ±0.168 | | | 24.32^e-g^ ±2.91 | | 44.02^d-g^ ±2.05 | | | 11.04^g^ ±0.48 | | 94.34^a^ ±0.69 | |
| 15 | Soltanieh | 5.37^b-e^± 0.64 | 7.36^b-e^ ± 1.1 | 8.6^bc^ ± 1.27 | | | 15.83^a^ ± 1.77 | | 0.57^ab^ ±0.11 | | | 1.23^ab^±0.48 | | 0.73^a-d^ ±0.205 | | 0.76^b-d^ ±0.042 | | | 22.76^f-g^ ±1.2 | | 67.1^a-c^ ±1.06 | | | 22.53^c-f^ ±2.21 | | 89.46^a^ ±3.6 | |
| 16 | Taft | 7.86^b^ ± 0.51 | 8.71^b-d^ ± 1.15 | 3.51^ef^ ± 0.59 | | | 10.64^a-d^ ± 1.45 | | 0.579^ab^±0.15 | | | 1.53^ab^±0.14 | | 0.32^d^ ±0.091 | | 1.09^bc^ ±0.067 | | | 30.84^b-f^ ±1.54 | | 56.19^a-e^ ±0.7 | | | 22.53^ef^ ±1.38 | | 60.47^bc^ ±1.43 | |

Table S2. Effect of Azotobacter and salinity interaction on the enzyme activities of different Iranian licorice accessions.

S stands for salinity (S_0_; no salinity application, and S_1_; 200 mM NaCl,) and A standsd for Azotobacter inoculation (A_0_; non-inoculation, and A_1_; inoculation by Azotobacter). Mean values with the same superscript within a column are not significantly different (*p* ≤ 0.05). Tukey test. Means± Standard error (SE).

Table S3. Analyze variance of Irainin licorice accessions biochemical traits understudied treatments.

| Source | Catalas | | | Superoxide dismutase | | | Peroxidase | | | Polyphenol oxidase | | | Ascorbate peroxidase | | | Phenylalanine ammonia-lyase | | |
| --- | --- | --- | --- | --- | --- | --- | --- | --- | --- | --- | --- | --- | --- | --- | --- | --- | --- | --- |
|  | df | F | P | df | F | P | df | F | P | df | F | P | df | F | P | df | F | P |
| Accession | 15 | 32.51 | 0.00 | 15 | 7.45 | 0.00 | 15 | 3.35 | 0.00 | 15 | 1.18 | 0.29 | 15 | 22.63 | 0.00 | 15 | 4.52 | 0.00 |
| Bacterial inoculation | 1 | 2.77 | 0.098 | 1 | 3.62 | 0.05 | 1 | 4.37 | 0.03 | 1 | 073 | 0.39 | 1 | 2.18 | 0.14 | 1 | 10.52 | 0.00 |
| Salinity | 1 | 55.96 | 0.00 | 1 | 24.05 | 0.00 | 1 | 4.01 | 0.04 | 1 | 9.67 | 0.00 | 1 | 320.6 | 0.00 | 1 | 11.26 | 0.00 |
| Accession×Bacterial inoculation | 15 | 7.19 | 0.00 | 15 | 3.49 | 0.00 | 15 | 1.57 | 0.09 | 15 | 0.62 | 0.85 | 15 | 23.89 | 0.00 | 15 | 1.85 | 0.03 |
| Accession×Salinity | 15 | 8.53 | 0.00 | 15 | 2.3 | 0.00 | 15 | 0.97 | 0.49 | 15 | 0.86 | 0.61 | 15 | 31.9 | 0.00 | 15 | 1.3 | 0.21 |
| Bacterial inoculation×Salinity | 1 | 35.66 | 0.00 | 1 | 11.47 | 0.00 | 1 | 10.6 | 0.00 | 1 | 7.67 | 0.00 | 1 | 98.87 | 0.00 | 1 | 13.89 | 0.00 |
| Accession×Bacterial inoculation×Salinity | 15 | 7.78 | 0.00 | 15 | 2.26 | 0.00 | 15 | 1.02 | 0.43 | 15 | 1.58 | 0.08 | 15 | 28.3 | 0.00 | 15 | 0.6 | 0.87 |
| Error | 128 | - | - | 128 | - | - | 128 | - | - | 128 | - | - | 128 | - | - | 128 | - | - |

Table S4. Analyze variance of Irainin licorice accessions biochemical traits understudied treatments.

| Source | Electrolyte leakage | | | Antioxidant activity | | | Hydrogen peroxide | | | Membrane stability index | | | Malondialdehyde | | |
| --- | --- | --- | --- | --- | --- | --- | --- | --- | --- | --- | --- | --- | --- | --- | --- |
|  | df | F | P | df | F | P | df | F | P | df | F | P | df | F | P |
| Accession | 15 | 2.36 | 0.00 | 15 | 13.67 | 0.00 | 15 | 63.20 | 0.00 | 15 | 2.29 | 0.00 | 15 | 5.21 | 0.00 |
| Bacterial inoculation | 1 | 1.23 | 0.27 | 1 | 4.61 | 0.03 | 1 | 17.94 | 0.00 | 1 | 1.11 | 0.29 | 1 | 2.57 | 0.11 |
| Salinity | 1 | 92.28 | 0.00 | 1 | 17.96 | 0.00 | 1 | 123.50 | 0.00 | 1 | 80.97 | 0.00 | 1 | 20.79 | 0.00 |
| Accession×Bacterial inoculation | 15 | 1.16 | 0.31 | 15 | 6.28 | 0.00 | 15 | 18.82 | 0.00 | 15 | 1.06 | 0.39 | 15 | 3.43 | 0.00 |
| Accession×Salinity | 15 | 1.89 | 0.03 | 15 | 2.87 | 0.00 | 15 | 20.46 | 0.00 | 15 | 1.78 | 0.04 | 15 | 3.43 | 0.00 |
| Bacterial inoculation×Salinity | 1 | 0.15 | 0.69 | 1 | 17.69 | 0.00 | 1 | 42.46 | 0.00 | 1 | 0.37 | 0.54 | 1 | 7.30 | 0.00 |
| Accession×Bacterial inoculation×Salinity | 15 | 0.94 | 0.52 | 15 | 1.73 | 0.05 | 15 | 19.54 | 0.00 | 15 | 0.83 | 0.63 | 15 | 2.46 | 0.00 |
| Error | 128 | - | - | 128 | - | - | 128 | - | - | 128 | - | - | 128 | - | - |

Table S5. Analyze variance of Irainin licorice accessions biochemical traits understudied treatments.

| Source | Plant height | | | Crown diameter | | |
| --- | --- | --- | --- | --- | --- | --- |
|  | df | F | P | df | F | P |
| Accession | 15 | 17.36 | 0.00 | 15 | 1.77 | 0.04 |
| Bacterial inoculation | 1 | 10.88 | 0.00 | 1 | 0.00 | 0.96 |
| Salinity | 1 | 4.00 | 0.48 | 1 | 3.66 | 0.05 |
| Accession×Bacterial inoculation | 15 | 1.66 | 0.06 | 15 | 1.00 | 0.45 |
| Accession×Salinity | 15 | 1.09 | 0.37 | 15 | 0.61 | 0.86 |
| Bacterial inoculation×Salinity | 1 | 6.10 | 0.01 | 1 | 0.79 | 0.37 |
| Accession×Bacterial inoculation×Salinity | 15 | 0.96 | 0.50 | 15 | 0.38 | 0.98 |
| Error | 128 | - | - | 128 | - | - |
